# Risks of *Brucella* spp. Infection in Dogs

**DOI:** 10.3390/pathogens12101209

**Published:** 2023-10-01

**Authors:** Valentina Virginia Ebani

**Affiliations:** 1 Department of Veterinary Sciences, University of Pisa, Viale delle Piagge 2, 56124 Pisa, Italy; valentina.virginia.ebani@unipi.it; 2 Centre for Climate Change Impact, University of Pisa, Via del Borghetto 80, 56124 Pisa, Italy

Dogs are known to be susceptible to different *Brucella* species, even though canine brucellosis is usually associated with *B. canis*. This pathogen causes reproductive disorders in bitches but is also responsible for inflammation of the male genital tract, with consequent infertility. Splenitis, nephritis, diskospondylitis, endocarditis, uveitis, and chorioretinitis have been observed in both genders [1]. 

*Brucella canis* is a zoonotic agent; therefore, it represents a severe threat not only for the canine health status, but also for owners, breeders, veterinarians, and kennel personnel. On the other hand, brucellosis can also be caused in dogs by other species, such as *B. abortus*, *B. melitensis* and *B. suis*. 

Recently, Girault and colleagues [2] reported two canine cases of infection by *B. suis* biovar 2. These cases were observed in France in 2020 and 2022, respectively, and both dogs involved were males. One dog had prostatitis and the other one had orchitis. Both animals had received no contact with pig farms, but they frequently strolled in the surrounding forests. 

This study highlights the possibility that dogs can be infected by *Brucella* species and biovars considered uncommon for them and shows the high risk of contracting infections in wild environments. 

Hunting dogs are often exposed to pathogens, bacteria, viruses and parasites excreted by infected wild animals. They also face contact with hematophagous arthropods harboring microorganisms able to cause canine diseases. In addition, hunting dogs are exposed to the risk of being attacked and injured by wild animals, primarily wild boars; this direct contact favors the entrance of pathogens through lesions. The ingestion and licking of infected animals’ carcasses and/or contaminated puddle water, and also the inhalation of contaminated dust, are the most common routes of infection by many pathogens, including brucellae. This is not only true for hunting dogs, but also for companion dogs frequenting wild environments with their owners for recreational activity. 

Different *Brucella* species circulate in the wild. The presence of *Brucella abortus* and *B. melitensis*, even though they are usually related to farm ruminants, has been reported in different wild mammal species [3,4]. *Brucella suis* has been found in different mammals in relation to its serovars and the geographic area. Biovars 1 and 3 have been isolated from feral pigs and peccaries (family Tayassuidae), biovar 2 from hares (*Lepus europaeus*) and Eurasian wild boars (*Sus scrofa*), biovar 4 from reindeers or caribou (*Rangifer tarandus* and its subspecies), and biovar 5 from small rodents [4]. 

*Brucella microti* has been isolated from voles (*Microtus arvalis*) [5], red foxes (*Vulpes vulpes*) [6], and wild boars [7]. *B. vulpis* has been detected in red foxes [8]. 

The pathogenicity of the more recently identified *Brucella* species, such as *B. microti* and *B. vulpis*, is still under study. However, it cannot be excluded they are able to affect dogs and humans, as well. 

The report by Girault et al. [2] is worthy of attention because the *B. suis* biovar 2 had never previously been isolated from domestic or wild canids, suggesting that other brucella species or biovars in addition to those reported in the scientific literature may affect dogs.

Although brucellosis has almost been eradicated in many countries, this infection still represents a severe threat for livestock, dogs and humans. In these areas, the diagnostic hypotheses often are not oriented towards *Brucella* infections, as happened in the two cases reported by Girault and colleagues.

In the view of this Issue, veterinarians should well know the epidemiological scenarios of the different pathogens and the diagnostic tests required in order to promptly and correctly act with therapeutic and preventive measures. In the case of suspected canine brucellosis, clinicians must request diagnosis for infection by all brucellae and not just *B. canis*. In this regard, they should remember that 1) at least two different serological tests are necessary because different antigens are employed when antibodies against smooth (*B. abortus*, *B. melitensis*, *B. suis*) or rough brucellae (*B. canis*) are investigated, and that 2) only the direct diagnosis of clinical specimens (e.g., semen, aborted fetus, vaginal discharge) will allow researchers to identify the *Brucella* species and biovar responsible for the infection case.
